# Extending an evidence hierarchy to include topics other than treatment: revising the Australian 'levels of evidence'

**DOI:** 10.1186/1471-2288-9-34

**Published:** 2009-06-11

**Authors:** Tracy Merlin, Adele Weston, Rebecca Tooher

**Affiliations:** 1 Adelaide Health Technology Assessment (AHTA), Discipline of Public Health, University of Adelaide, Adelaide, South Australia, Australia; 2 Health Technology Analysts, Balmain, New South Wales, Australia; 3 Discipline of Obstetrics and Gynaecology, University of Adelaide, Adelaide, South Australia, Australia

## Abstract

**Background:**

In 1999 a four-level hierarchy of evidence was promoted by the National Health and Medical Research Council in Australia. The primary purpose of this hierarchy was to assist with clinical practice guideline development, although it was co-opted for use in systematic literature reviews and health technology assessments. In this hierarchy *interventional *study designs were ranked according to the likelihood that bias had been eliminated and thus it was not ideal to assess studies that addressed other types of clinical questions. This paper reports on the revision and extension of this evidence hierarchy to enable broader use within existing evidence assessment systems.

**Methods:**

A working party identified and assessed empirical evidence, and used a commissioned review of existing evidence assessment schema, to support decision-making regarding revision of the hierarchy. The aim was to retain the existing evidence levels I-IV but increase their relevance for assessing the quality of individual diagnostic accuracy, prognostic, aetiologic and screening studies. Comprehensive public consultation was undertaken and the revised hierarchy was piloted by individual health technology assessment agencies and clinical practice guideline developers. After two and a half years, the hierarchy was again revised and commenced a further 18 month pilot period.

**Results:**

A suitable framework was identified upon which to model the revision. Consistency was maintained in the hierarchy of "levels of evidence" across all types of clinical questions; empirical evidence was used to support the relationship between study design and ranking in the hierarchy wherever possible; and systematic reviews of lower level studies were themselves ascribed a ranking. The impact of ethics on the hierarchy of study designs was acknowledged in the framework, along with a consideration of how harms should be assessed.

**Conclusion:**

The revised evidence hierarchy is now widely used and provides a common standard against which to initially judge the likelihood of bias in individual studies evaluating interventional, diagnostic accuracy, prognostic, aetiologic or screening topics. Detailed quality appraisal of these individual studies, as well as grading of the body of evidence to answer each clinical, research or policy question, can then be undertaken as required.

## Background

The corner-stone of evidence-based healthcare and health technology assessment is critical appraisal of the evidence underpinning a finding. Different methods are available for assessing the quality of the evidence, including ranking the body of evidence according to a hierarchy which indicates the level of bias associated with the different study designs that have contributed to the evidence-base. In Australia, the standard evidence hierarchy in use since 1999 has been the National Health and Medical Research Council (NHMRC) Designation of Levels of Evidence [1]. This hierarchy ranks the body of evidence into four levels – from systematic reviews of randomised trials at the top of the hierarchy, to case series and case reports at the bottom of the hierarchy (Table 1). Its intended purpose was to summarise the body of evidence for interventions (eg treatment effectiveness). Through widespread use in clinical practice guideline development and health technology assessment, it became increasingly clear that: i) the hierarchy was being used to address research questions that did not relate to interventions; ii) the hierarchy – which is primarily concerned with the association between bias and study design characteristics – was being relied upon for the entire evidence appraisal rather than there being a standardised appraisal of study quality as suggested [2]; and iii) that although the aim was to use the hierarchy to summarise the entire body of evidence – this was occurring rather haphazardly in practice.

**Table 1:** Designations of levels of evidence [1]

Level of evidence	Study design
I	Evidence obtained from a systematic review of all relevant randomised controlled trials
II	Evidence obtained from at least one properly-designed randomised controlled trial
III-1	Evidence obtained from well-designed pseudorandomised controlled trials (alternate allocation or some other method)
III-2	Evidence obtained from comparative studies (including systematic reviews of such studies) with concurrent controls and allocation not randomised, cohort studies, case-control studies, or interrupted time series with a control group
III-3	Evidence obtained from comparative studies with historical control, two or more single arm studies, or interrupted time series without a parallel control group
IV	Evidence obtained from case series, either post-test or pre-test/post-test

This paper describes the first stage of developing a hierarchy to rank the quality of *individual *study designs to address different types of questions. The second stage of developing or adapting a simple, intuitive system to grade the entire *body of evidence *is discussed elsewhere [3,4], and will be the subject of a forthcoming publication.

### The existing hierarchy

The existing NHMRC hierarchy of evidence was developed as part of a comprehensive series of handbooks which outlined the methods for evaluating evidence and developing and disseminating clinical practice guidelines [1,2,5-9].

These handbooks recommended that the body of evidence should be assessed along three dimensions: strength, size of effect and clinical relevance. In this schema the strength of evidence was determined by the level of evidence, the quality of the evidence and its statistical precision. It was further assumed that the results from a 'body of evidence' could be distilled down to a single size of effect, with associated statistical precision and that the clinical relevance of this result could be determined eg a pooled relative risk and confidence interval obtained through meta-analysis. The *evidence level*, designated according to the hierarchy (Table 1), assessed the likelihood that the 'body of evidence' producing this single size of effect was affected by bias.

It became clear on applying this schema that the available evidence-base for clinical practice guidelines and health technology assessments was often not amenable to meta-analysis. Thus *statistical synthesis *for each of the outcomes of interest into one estimate of effect, with associated statistical precision and determination of clinical relevance, was often not possible. As a consequence, in practice, the dimensions of evidence were often applied to *individual *studies and were complemented with a *narrative synthesis *of the overall findings from the body of evidence. The difficulty with this approach was that the original hierarchy of evidence was not designed, nor worded, to refer to the strength of the evidence obtained from individual studies.

Further, the hierarchy was designed to assess evidence from intervention studies that evaluated therapeutic effectiveness. It was therefore not appropriate for assessing studies addressing diagnostic accuracy, aetiology, prognosis or screening interventions. The study designs best suited to answer these types of questions are not always the same, or presented in the same order, as that given in the original NHMRC hierarchy of evidence. It was clear that an alternative approach to appraising evidence was needed.

The NHMRC therefore created a working party of clinical practice guideline developers, health technology assessment producers and methodologists (the Working Party) to develop a revised hierarchy of evidence for individual studies (first stage) which addressed these issues, as well as a method for appraising the body of evidence (second stage) that could be used by guideline developers and others.

The objective of the first stage was to create a framework that aligned as closely as possible with the original evidence hierarchy – to minimise confusion for current users and maintain consistency with previous use of the hierarchy – but which could also rank individual studies addressing questions other than therapeutic effectiveness. Due consideration was to be given to methods used by other organisations to develop "levels of evidence", in order to minimise duplication of effort.

## Methods

Recognising the need for an updated hierarchy of evidence, a review was conducted of existing frameworks for assessing non-randomised and non-interventional evidence that are used by Health Technology Assessment (HTA) agencies and guideline developers world-wide [10]. This internal report commissioned by the NHMRC, and conducted by HTanalysts, provided a resource for the NHMRC and the Working Party to enable revision of the current hierarchy of evidence. The aim was to adapt, if possible, an existing evidence hierarchy or hierarchies.

The report searched for comprehensive evidence frameworks that incorporated non-intervention evidence via HTA and Guideline group websites that were identified through the membership of the International Network of Agencies for Health Technology Assessment (INAHTA) and the Guidelines International Network (GIN) (see Appendix). Bibliographies of identified publications were examined and targeted Medline/EMBASE searches were conducted. Frameworks were included if they were published in English, were developed by a reputable HTA or guideline agency, and contained guidance on at least one of the methodological processes involved in undertaking an evidence-based assessment (Guideline, HTA or systematic review).

The identified frameworks were then used to inform the revision of the NHMRC evidence hierarchy. Six key factors were considered integral to this revision process, specifically that:

1. the hierarchy addressed all types of questions and was not limited to treatment effectiveness alone;

2. the levels I-IV were maintained and aligned as closely as possible with the current NHMRC (treatment effectiveness) hierarchy;

3. the hierarchy related to individual studies rather than a body of evidence (given a multi-factorial method of "grading" the body of evidence was being developed/adapted concurrently via the NHMRC Working Party);

4. the hierarchy remained broadly consistent across types of question;

5. empirical evidence supported the placement of a particular study design in the evidence hierarchy wherever possible – that is, the relationship between study design and bias for each clinical or research question had been assessed empirically; or if not, there were good theoretical grounds for such placement in the hierarchy; and

6. subjective terms regarding the "quality" of studies eg "well designed", "properly designed" would be removed. The level of evidence would be assessed on the basis of study design characteristics alone. Determination of the overall "quality" of the study would be independently determined using appropriate – and validated, where possible – checklists suitable for each study design and question.

The "Levels" subgroup of the Working Party addressed each of these criteria while drafting a revision of the evidence hierarchy. This first iteration of the hierarchy was slightly modified after consultation with other methodological experts within the wider Working Party. A second iteration of the hierarchy was presented to Australian and New Zealand evaluators undertaking health technology assessments for the Australian Medical Services Advisory Committee (MSAC). Other international experts on evidence appraisal were contacted and provided feedback on the hierarchy. These suggestions were discussed and some substantial revisions – particularly concerning the diagnostic accuracy evidence hierarchy – were incorporated into a version of the hierarchy that was suitable for piloting.

The hierarchy was piloted by NHMRC clinical practice guideline developers and health technology assessment evaluation groups in Australia and New Zealand from November 2004 until June 2007. Public consultation throughout this period was invited through the medium of international conferences and workshops – specifically the Cochrane Colloquium and the Health Technology Assessment international (HTAi) conference [11-13] – and through the NHMRC website. With the website, a feedback form allowing free text responses to a series of questions regarding the utility and adaptability of the revised hierarchy was provided, along with a section for suggested methods for improving the hierarchy. The hierarchy was amended and a further pilot stage was then conducted from February 2008 to February 2009. In total, approximately a dozen responses were submitted through the website, predominantly by individuals or organisations that had trialled the new evidence hierarchy.

## Results

### Identifying possible frameworks for adaptation

The 2004 report commissioned by the NHMRC identified 18 evidence frameworks that were relevant for clinical evaluation of non-interventional evidence at that time [10]. Three of the evidence evaluation frameworks were found to use a hierarchy that related to questions other than treatment or intervention effectiveness. The National Institute for Clinical Excellence (NICE) provided a hierarchy that used levels of evidence for assessment of therapeutic effectiveness (based on those developed by the Scottish Intercollegiate Guidelines Network – SIGN) as well as for diagnostic accuracy [14]. The National Health Service Centre for Reviews and Dissemination (NHS CRD) used a framework that included levels of evidence for assessing questions of effectiveness, diagnostic accuracy, and efficiency [15]. Finally, the Centre for Evidence Based Medicine (CEBM) hierarchy included levels of evidence for assessing questions of therapy/prevention and aetiology/harm, prognosis, diagnosis, differential diagnosis/symptom prevalence, and economic and decision analyses [16].

In terms of addressing different types of questions, the CEBM framework was found to be the most comprehensive and a suitable evidence hierarchy upon which to model the revised NHMRC hierarchy of evidence, although all three provided useful information.

### The revised NHMRC hierarchy

Each of the six key factors considered integral to a revised NHMRC evidence hierarchy were adopted. Five separate research areas were addressed – interventions, diagnostic accuracy, prognosis, aetiology and screening.

A greatly expanded table was created, largely based on the design of the CEBM framework, which included five separate columns for each of the different research areas (see Additional file 1). However, even though the CEBM layout was very closely followed in the revised NHMRC hierarchy, the number of research questions addressed and description of studies did differ markedly from the CEBM framework. Empirical evidence of study design biases and epidemiological theory were used to rank the study designs within each research area. It was suggested that when referring to studies designated a level of evidence according to the revised NHMRC hierarchy, both the level and corresponding research area or question should be used eg. level II intervention evidence; level IV diagnostic evidence; level III-2 prognostic evidence.

To support users of the revised NHMRC evidence hierarchy, explanatory notes (see Additional file 1) and a glossary of study designs and terminology (see Additional file 2) were developed and adapted from the NHMRC handbooks [1,2,5-9]. The explanatory notes provide the context for the evidence hierarchy, with guidance on how to apply and present the levels of evidence. The glossary provides a definition of each of the given study designs.

## Discussion

The revised NHMRC hierarchy of evidence largely addresses the issues which brought about its development. This hierarchy was developed using a combination of evidence, theory and consultation. The Working Party was able to successfully achieve its aim of providing a practical and usable tool for evidence-based healthcare practitioners and researchers. A number of special considerations were addressed in the development of this revised hierarchy, and some limitations were acknowledged when designing the hierarchy.

## Limitations

The evidence-base underpinning the development of a hierarchy such as this is limited. For intervention research questions there were some studies and a systematic review showing the degree of bias associated with observational and non-randomised studies, in comparison to randomised controlled trials [17-19]. However, for diagnostic research questions, at the time of developing the hierarchy we were aware of only one study on design-related bias associated with diagnostic studies [20]. In instances where the evidence was lacking to determine placement of the study design in the hierarchy, the CEBM evidence framework was used, along with epidemiology texts [21] and consensus expert opinion.

An evidence hierarchy addressing individual studies, alone, cannot provide interpretation of the results of a 'body of evidence' and the various contextual factors that can impinge on the interpretation of results (eg external validity/applicability). The 'Working Party' believes that any assessment of evidence underpinning a question involves three steps:

1. determine the level of evidence of individual studies addressing that question and rank the evidence accordingly;

2. appraise the quality of the evidence within each ranking using basic clinical epidemiology and biostatistical principles outlined in widely available critical appraisal checklists and tools; and

3. synthesise the findings from steps 1 and 2 and give greatest weight to the highest quality/highest ranked evidence. After including consideration of contextual factors, make a clear and transparent decision or recommendation regarding the strength and applicability of the findings from the body of evidence, and grade that recommendation.

Steps 1 and 2 are addressed in this paper. Step 3 was undertaken by the NHMRC Working Party through creating a process and system for classifying and grading the body of evidence that takes into account dimensions other than the internal validity of the studies – an issue which has received similar attention in other countries [22,23]. Progress on other grading systems to date has primarily centred on therapeutic safety and effectiveness research questions [24,25], although there have been recent moves towards explicitly incorporating diagnostic evidence [26]. The NHMRC Working Party has developed a multi-dimensional system to grade the evidence and develop recommendations in a user-friendly manner but which also addresses various types of research question (through use of this revised NHMRC evidence hierarchy as an intermediary step). This "grading" process is reported elsewhere and will be the subject of a subsequent publication [3,4].

While the revised hierarchy described in this paper has greatly expanded the types of studies that can be assigned a level of evidence, it does not cover qualitative research or economic analysis. There are existing hierarchies of evidence for economic analysis, although it is unclear if the methodological basis for the ranking within these hierarchies is supported by evidence and theory [15,16]. Should there be an expressed need to expand the revised NHMRC hierarchy to include economic analysis, this can occur when the NHMRC handbooks are updated.

Methods for synthesising qualitative research evidence are still being developed by groups such as the Cochrane Collaboration [27] and others [28,29]. In this context, critical appraisal guides and hierarchies of qualitative evidence have begun to appear in the literature [30]. A proper consideration of these issues was beyond the scope of this project and outside the methodological expertise of the Working Party. However, this should be addressed by investigators with appropriate expertise in qualitative research methods as part of the NHMRC handbook updates.

### Special considerations

#### 1. Systematic reviews of lower level evidence

In general, the Working Party took the view that systematic reviews should only be assigned a level of evidence as high as the studies contained therein. Even the best quality systematic reviews will still only be able to answer a research question on the basis of the evidence it has collated and synthesised. Thus any overall conclusions will be affected by the internal validity of the primary research evidence included. However, consistent with the original NHMRC hierarchy of evidence, Level I of the revised hierarchy was retained as a systematic review of all relevant level II studies, recognising that meta-analysis of Level II studies can increase the precision of the findings of individual Level II studies [31].

#### 2. Studies of diagnostic test accuracy

The effectiveness of a diagnostic test or a screening test requires either direct evidence ie the impact of the test on patient health outcomes (outlined in the 'Intervention' and 'Screening' columns, respectively, in the revised hierarchy) [26] or, if certain conditions are fulfilled, the linking of evidence of diagnostic test accuracy (assessed using the 'Diagnostic accuracy' column in the hierarchy) with evidence of change in management and the likely effect of that change in management on patient health outcomes (assessed using the 'Intervention' column in the revised hierarchy) [32,33].

The development of levels of evidence for studies of diagnostic accuracy proved to be more difficult than for the other types of research question. In studies of diagnostic accuracy the basic study design is cross-sectional, in which all participants receive both the index test and the reference standard. In order to rank the validity of each individual study's results it was found that a more specific discussion of study design was required. To aid with the interpretation and ranking of studies comprehensive explanatory notes were developed. To some extent the degree of bias introduced by a particular study design feature is dependent upon both the disease and the diagnostic test context under investigation. Well-developed critical appraisal skills of the reviewers of diagnostic test interventions are therefore essential. Methods for assessing diagnostic test accuracy by systematic review and meta-analysis have been progressing over a relatively short period of time (especially compared with studies of therapeutic effectiveness) [34-37]. As this methodology matures, the descriptive nature of the 'Diagnostic accuracy' levels in the revised hierarchy may no longer be required, as study designs in which bias is minimised are recognised (and possibly even named) as is currently the case with studies of therapeutic effectiveness.

#### 3. Correct classification of the research question

One other difficulty has been noted with use of the evidence hierarchy. The difficulty is not with the study designs or the ranking of the study designs, but rather with distinguishing between an aetiological and prognostic research question – and thus correct use of the relevant hierarchy. Both aetiology and prognosis relate to an identification of risk factors for an outcome and so the relevant study designs are quite similar. The key when determining if a research question is aetiological or prognostic is to identify the population of interest. For prognostic questions, all the population has the condition/disease and the aim is to determine what factors will predict an outcome for that population (eg survival) [2]. For example, "What are the risk factors for suicide in adolescent depression?" These factors can be causal (eg a treatment modality), effect modifiers (eg age) or just associations or markers. For aetiology questions, the key is ensuring the population of interest do not or did not have the condition/disease at some point in time, so that causality of the risk factor can be determined [2]. For example, "What are the risk factors for adolescent depression?" The explanatory notes to the hierarchy cannot make this distinction between aetiology and prognosis completely clear because of the degree of overlap in the relevant study designs.

#### 4. Assessment of study quality

The revised hierarchy of evidence is intended to be used as just one component in determining the strength of the evidence; that is, determining the likelihood of bias from the study design alone. This component is seen as a broad indicator of likely bias and can be used to roughly rank individual studies within a body of evidence. However, study quality within each of the levels of evidence needs to be assessed more rigorously. The Working Party believes that there are so many factors affecting the internal validity of study results (e.g. bias, confounding, results occurring by chance, impact of drop-outs), with different factors affecting different study designs, that a proper assessment of study quality can only occur with the use of an appropriate and/or validated checklist suitable for each study design or research question [2,15,25,37,38]. In the accompanying documentation to the revised evidence hierarchy, suggestions have been made as to the appropriate checklists for a formal critical appraisal of studies addressing the different types of research question [4].

#### 5. Ethical considerations

The impact of ethics on the hierarchy of study designs was acknowledged in the revised evidence hierarchy. Separate columns for aetiology and intervention research questions were produced in order to address trial feasibility and ethical issues. Explanatory notes appended to the hierarchy indicate that if it is possible and/or ethical to determine a causal relationship using experimental evidence, then the 'Intervention' hierarchy of evidence should be used. However, if it is only possible and/or ethical to determine a causal relationship using observational evidence (for example if it is not ethical to allocate groups to a potentially harmful exposure such as nuclear radiation), then the 'Aetiology' hierarchy of evidence should be used [39,40]. In the latter scenario, the highest level of evidence that could be used to address the question would be observational and not experimental.

#### 6. Assessment of harms/safety

There is guidance in the explanatory notes about how to deal with the evaluation of comparative harms and safety in the research area of interest. Assessment of comparative harms/safety should occur according to the hierarchy presented for each of the research questions, with the proviso that this assessment occurs within the context of the topic being assessed. Some harms (as well as some effectiveness outcomes) are rare and cannot feasibly be captured within randomised controlled trials [41,42], in which case lower levels of evidence may be the only type of evidence that is practically achievable; physical harms and psychological harms may need to be addressed by different study designs [43]; harms from diagnostic testing include the likelihood of false positive and false negative results [44,45]; harms from screening include the likelihood of false alarm and false reassurance results [46].

No single evidence-framework can address all of the safety and effectiveness issues associated with different research areas. The aim of the explanatory note was to explicitly recognise that these differences will occur and to adapt the hierarchy where necessary.

## Conclusion

Given the extensive pilot process – four years – this new evidence hierarchy is now the standard for judging "levels of evidence" for the purposes of health technology assessment and clinical practice guideline development in Australia.

Although this broad ranking tool for assessing study quality is intended for use as an intermediary step within the new NHMRC system to grade the body of evidence addressing a clinical, research or policy question [4], it can be applied within existing grading systems eg GRADE [47], SIGN [25] with the benefit of allowing a ranking of evidence that addresses research questions or areas other than therapeutic effectiveness.

This tool is particularly advantageous for structuring a narrative meta-synthesis of results in an evidence report or health technology assessment. Studies and study results can initially be ranked by study design (evidence level) using the revised evidence hierarchy, and then be further ranked *within *each evidence level with the use of appropriate and validated quality appraisal checklists. A grading of the body of evidence can then be applied, if relevant.

## Competing interests

Meeting attendance fees for this methodological work were paid to the authors by the National Health and Medical Research Council (NHMRC), a not-for-profit research organisation funded by the Australian Government Department of Health and Ageing. One of the functions of the NHMRC is to develop and disseminate health publications to health professionals and consumers in Australia. They produce health advisories, evidence-based clinical practice guidelines and methodology publications.

## Authors' contributions

TM instigated the revision of the original NHMRC evidence hierarchy, co-developed the revised evidence hierarchy, wrote the explanatory notes and glossary, drafted the manuscript, and incorporated the feedback received on both the hierarchy and the manuscript. AW conducted the review of international frameworks assessing non-randomised or non-interventional evidence (in conjunction with Dr Kristina Coleman and Dr Sarah Norris), co-developed the revised evidence hierarchy, and contributed to the development of the manuscript. RT co-developed the revised evidence hierarchy and contributed to the development of the manuscript. All authors read and approved the final manuscript.

## Appendix

Searches were conducted in June 2004. Enquiries regarding the search strategies should be directed to the Evidence Translation Section, National Health and Medical Research Council, Canberra, ACT, Australia.

## Pre-publication history

The pre-publication history for this paper can be accessed here:

http://www.biomedcentral.com/1471-2288/9/34/prepub

## Supplementary Material

Additional file 1 **Additional File **1**NHMRC Evidence Hierarchy: designations of 'levels of evidence' according to type of research question (including explanatory notes)**. Revised NHMRC evidence hierarchy and explanatory notes. Click here for file

Additional file 2 **Additional File **2**Study design glossary (alphabetic order)**. Description of study designs included in the revised NHMRC evidence hierarchy. Click here for file
