# Supplementary material for: Extending an evidence hierarchy to include topics other than treatment: revising the Australian 'levels of evidence'
Source: BMC Med Res Methodol. 2009 Jun 11;9:34. doi: 10.1186/1471-2288-9-34 (PMC2700132; doi:10.1186/1471-2288-9-34)
Supplement: Additional file 1 — NHMRC Evidence Hierarchy: designations of 'levels of evidence' according to type of research question (including explanatory notes). Revised NHMRC evidence hierarchy and explanatory notes. [file 1471-2288-9-34-S1.doc]

**Additional File 1 NHMRC Evidence Hierarchy: designations of ‘levels of evidence’ according to type of research question** (including explanatory notes)

| **Level** | **Intervention 1** | **Diagnostic accuracy 2** | **Prognosis** | **Aetiology 3** | **Screening Intervention** |
| --- | --- | --- | --- | --- | --- |
| I 4 | A systematic review of level II  studies | A systematic review of level  II studies | A systematic review of level II studies | A systematic review of level II studies | A systematic review of level II studies |
| II | A randomised controlled trial | A study of test accuracy with: an independent, blinded comparison with a valid reference standard,5 among consecutive persons with a defined clinical presentation^6^ | A prospective cohort study^7^ | A prospective cohort study | A randomised controlled trial |
| III-1 | A pseudorandomised controlled trial  (i.e. alternate allocation or some other method) | A study of test accuracy with: an independent, blinded comparison with a valid reference standard,5 among non-consecutive persons with a defined clinical presentation^6^ | All or none^8^ | All or none^8^ | A pseudorandomised controlled trial  (i.e. alternate allocation or some other method) |
| III-2 | A comparative study with concurrent controls:  ▪ Non-randomised, experimental trial^9^  ▪ Cohort study  ▪ Case-control study  ▪ Interrupted time series with a control group | A comparison with reference standard that does not meet the criteria required for  Level II and III-1 evidence | Analysis of prognostic factors amongst persons in a single arm of a randomised controlled trial | A retrospective cohort study | A comparative study with concurrent controls:  ▪ Non-randomised, experimental trial  ▪ Cohort study  ▪ Case-control study |
| III-3 | A comparative study without concurrent controls:  ▪ Historical control study  ▪ Two or more single arm study^10^  ▪ Interrupted time series without a parallel control group | Diagnostic case-control study^6^ | A retrospective cohort study | A case-control study | A comparative study without concurrent controls:  ▪ Historical control study  ▪ Two or more single arm study |
| IV | Case series with either post-test or pre-test/post-test outcomes | Study of diagnostic yield (no reference standard)^11^ | Case series, or cohort study of persons at different stages of disease | A cross-sectional study or case series | Case series |

### Explanatory notes

1 Definitions of these study designs are provided on pages 7-8 *How to use the evidence: assessment and application of scientific evidence* (NHMRC 2000b*)* and in the accompanying Glossary.

**2** These levels of evidence apply only to studies of assessing the accuracy of diagnostic or screening tests. To assess the overall effectiveness of a diagnostic test there also needs to be a consideration of the impact of the test on patient management and health outcomes (Medical Services Advisory Committee 2005, Sackett and Haynes 2002). The evidence hierarchy given in the ‘Intervention’ column should be used when assessing the impact of a diagnostic test on health outcomes relative to an existing method of diagnosis/comparator test(s). The evidence hierarchy given in the ‘Screening’ column should be used when assessing the impact of a screening test on health outcomes relative to no screening or alternative screening methods.

3 If it is possible and/or ethical to determine a causal relationship using experimental evidence, then the ‘Intervention’ hierarchy of evidence should be utilised. If it is only possible and/or ethical to determine a causal relationship using observational evidence (eg. cannot allocate groups to a potential harmful exposure, such as nuclear radiation), then the ‘Aetiology’ hierarchy of evidence should be utilised.

4 A systematic review will only be assigned a level of evidence as high as the studies it contains, excepting where those studies are of level II evidence. Systematic reviews of level II evidence provide more data than the individual studies and any meta-analyses will increase the precision of the overall results, reducing the likelihood that the results are affected by chance. Systematic reviews of lower level evidence present results of likely poor internal validity and thus are rated on the likelihood that the results have been affected by bias, rather than whether the systematic review itself is of good quality. Systematic review *quality* should be assessed separately. A systematic review should consist of at least two studies. In systematic reviews that include different study designs, the overall level of evidence should relate to each individual outcome/result, as different studies (and study designs) might contribute to each different outcome.

5 The validity of the reference standard should be determined in the context of the disease under review. Criteria for determining the validity of the reference standard should be pre-specified. This can include the choice of the reference standard(s) and its timing in relation to the index test. The validity of the reference standard can be determined through quality appraisal of the study (Whiting et al 2003).

6 Well-designed population based case-control studies (eg. population based screening studies where test accuracy is assessed on all cases, with a random sample of controls) do capture a population with a representative spectrum of disease and thus fulfil the requirements for a valid assembly of patients. However, in some cases the population assembled is not representative of the use of the test in practice. In diagnostic case-control studies a selected sample of patients already known to have the disease are compared with a separate group of normal/healthy people known to be free of the disease. In this situation patients with borderline or mild expressions of the disease, and conditions mimicking the disease are excluded, which can lead to exaggeration of both sensitivity and specificity. This is called spectrum bias or spectrum effect because the spectrum of study participants will not be representative of patients seen in practice (Mulherin and Miller 2002).

7 At study inception the cohort is either non-diseased or all at the same stage of the disease. A randomised controlled trial with persons either non-diseased or at the same stage of the disease in *both* arms of the trial would also meet the criterion for this level of evidence.

8 All or none of the people with the risk factor(s) experience the outcome; and the data arises from an unselected or representative case series which provides an unbiased representation of the prognostic effect. For example, no smallpox develops in the absence of the specific virus; and clear proof of the causal link has come from the disappearance of small pox after large-scale vaccination.

9 This also includes controlled before-and-after (pre-test/post-test) studies, as well as adjusted indirect comparisons (ie. utilise A vs B and B vs C, to determine A vs C with statistical adjustment for B).

10 Comparing single arm studies ie. case series from two studies. This would also include unadjusted indirect comparisons (ie. utilise A vs B and B vs C, to determine A vs C but where there is no statistical adjustment for B).

11 Studies of diagnostic yield provide the yield of diagnosed patients, as determined by an index test, without confirmation of the accuracy of this diagnosis by a reference standard. These may be the only alternative when there is no reliable reference standard.

**Note A:** Assessment of comparative harms/safety should occur according to the hierarchy presented for each of the research questions, with the proviso that this assessment occurs within the context of the topic being assessed. Some harms (and other outcomes) are rare and cannot feasibly be captured within randomised controlled trials, in which case lower levels of evidence may be the only type of evidence that is practically achievable; physical harms and psychological harms may need to be addressed by different study designs; harms from diagnostic testing include the likelihood of false positive and false negative results; harms from screening include the likelihood of false alarm and false reassurance results.

**Note B:** When a level of evidence is attributed in the text of a document, it should also be framed according to its corresponding research question eg. level II intervention evidence; level IV diagnostic evidence; level III-2 prognostic evidence.

**Note C:** Each individual study that is attributed a “level of evidence” should be rigorously appraised using validated or commonly used checklists or appraisal tools to ensure that factors other than study design have not affected the validity of the results.

**Source:** Hierarchies adapted and modified from: NHMRC 1999; Bandolier 1999; Lijmer et al. 1999; Phillips et al. 2001 (see Additional File 2).
