# Supplementary material for: Extending an evidence hierarchy to include topics other than treatment: revising the Australian 'levels of evidence'
Source: BMC Med Res Methodol. 2009 Jun 11;9:34. doi: 10.1186/1471-2288-9-34 (PMC2700132; doi:10.1186/1471-2288-9-34)
Supplement: Additional file 2 — Study design glossary (alphabetic order). Description of study designs included in the revised NHMRC evidence hierarchy. [file 1471-2288-9-34-S2.doc]

**Additional File 2**

**STUDY DESIGN GLOSSARY (alphabetic order)**

*Adapted from NHMRC 2000ab, Glasziou et al 2001, Elwood 1998*

*Note: This is a specialised glossary that relates specifically to the study designs mentioned in the NHMRC Evidence Hierarchy. Glossaries of terms that relate to wider epidemiological concepts and evidence based medicine are also available – see* [*http://www.inahta.org/HTA/Glossary/*](http://www.inahta.org/HTA/Glossary/)*;* [*http://www.ebmny.org/glossary.html*](http://www.ebmny.org/glossary.html)

**All or none** – all or none of a series of people (case series) with the risk factor(s) experience the outcome. The data should relate to an unselected or representative case series which provides an unbiased representation of the prognostic effect. For example, no smallpox develops in the absence of the specific virus; and clear proof of the causal link has come from the disappearance of small pox after large scale vaccination. This is a rare situation.

**A study of test accuracy with: an independent, blinded comparison with a valid reference standard, among consecutive patients with a defined clinical presentation** – a cross-sectional study where a consecutive group of people from an appropriate (relevant) population receive the test under study (index test) and the reference standard test. The index test result is not incorporated in (is independent of) the reference test result/final diagnosis. The assessor determining the results of the index test is blinded to the results of the reference standard test and vice versa.

**A study of test accuracy with: an independent, blinded comparison with a valid reference standard, among non-consecutive patients with a defined clinical presentation** – a cross- sectional study where a non-consecutive group of people from an appropriate (relevant) population receive the test under study (index test) and the reference standard test. The index test result is not incorporated in (is independent of) the reference test result/final diagnosis. The assessor determining the results of the index test is blinded to the results of the reference standard test and vice versa.

**Adjusted indirect comparisons** – an adjusted indirect comparison compares single arms from two or more interventions from two or more separate studies via the use of a common reference ie A versus B and B versus C allows a comparison of A versus C when there is statistical adjustment for B. This is most commonly done in meta-analyses (see Bucher et al 1997). Such an indirect comparison should only be attempted when the study populations, common comparator/reference, and settings are very similar in the two studies (Song et al 2000).

**Case-control study** – people with the outcome or disease (cases) and an appropriate group of controls without the outcome or disease (controls) are selected and information obtained about their previous exposure/non-exposure to the intervention or factor under study.

**Case series** – a single group of people exposed to the intervention (factor under study).

**Post-test** – only outcomes after the intervention (factor under study) are recorded in the series of people, so no comparisons can be made.

**Pre-test/post-test** – measures on an outcome are taken before and after the intervention is introduced to a series of people and are then compared (also known as a ‘before- and-after study’).

**Cohort study** – outcomes for groups of people observed to be exposed to an intervention, or the factor under study, are compared to outcomes for groups of people not exposed.

**Prospective cohort study** – where groups of people (cohorts) are observed at a point in time to be exposed or not exposed to an intervention (or the factor under study) and then are followed prospectively with further outcomes recorded as they happen.

**Retrospective cohort study** – where the cohorts (groups of people exposed and not exposed) are defined at a point of time in the past and information collected on subsequent outcomes, eg. the use of medical records to identify a group of women using oral contraceptives five years ago, and a group of women not using oral contraceptives, and then contacting these women or identifying in subsequent medical records the development of deep vein thrombosis.

**Cross-sectional study** – a group of people are assessed at a particular point (or cross-section) in time and the data collected on outcomes relate to that point in time ie proportion of people with asthma in October 2004. This type of study is useful for hypothesis-generation, to identify whether a risk factor is associated with a certain type of outcome, but more often than not (except when the exposure and outcome are stable eg. genetic mutation and certain clinical symptoms) the causal link cannot be proven unless a time dimension is included.

**Diagnostic (test) accuracy** – in diagnostic accuracy studies, the outcomes from one or more diagnostic tests under evaluation (the *index test*/s) are compared with outcomes from a *reference standard test*. These outcomes are measured in individuals who are suspected of having the condition of interest. The term *accuracy* refers to the amount of agreement between the index test and the reference standard test in terms of outcome measurement. Diagnostic accuracy can be expressed in many ways, including sensitivity and specificity, likelihood ratios, diagnostic odds ratio, and the area under a receiver operator characteristic (ROC) curve (Bossuyt et al 2003)

**Diagnostic case-control study** – the index test results for a group of patients already known to have the disease (through the reference standard) are compared to the index test results with a separate group of normal/healthy people known to be free of the disease (through the use of the reference standard). In this situation patients with borderline or mild expressions of the disease, and conditions mimicking the disease are excluded, which can lead to exaggeration of both sensitivity and specificity. This is called spectrum bias because the spectrum of study participants will not be representative of patients seen in practice. *Note: this does not apply to well-designed population based case-control studies.*

**Historical control study** – outcomes for a prospectively collected group of people exposed to the intervention (factor under study) are compared with either (1) the outcomes of people treated at the same institution prior to the introduction of the intervention (ie. control group/usual care), or (2) the outcomes of a previously published series of people undergoing the alternate or control intervention.

**Interrupted time series with a control group** – trends in an outcome or disease are measured over multiple time points before and after the intervention (factor under study) is introduced to a group of people, and then compared to the outcomes at the same time points for a group of people that do not receive the intervention (factor under study).

**Interrupted time series without a parallel control group** – trends in an outcome or disease are measured over multiple time points before and after the intervention (factor under study) is introduced to a group of people, and compared (as opposed to being compared to an external control group).

**Non-randomised, experimental trial** - the unit of experimentation (eg. people, a cluster of people) is allocated to either an intervention group or a control group, using a non-random method (such as patient or clinician preference/availability) and the outcomes from each group are compared.

This can include:

(1) **a controlled before-and-after study**, where outcome measurements are taken before and after the intervention is introduced, and compared at the same time point to outcome measures in the control group.

(2) **an adjusted indirect comparison**, where two randomised controlled trials compare different interventions to the same comparator ie. the placebo or control condition. The outcomes from the two interventions are then compared indirectly. *See entry on adjusted indirect comparisons.*

**Pseudo-randomised controlled trial** - the unit of experimentation (eg. people, a cluster of people) is allocated to either an intervention (the factor under study) group or a control group, using a pseudo-random method (such as alternate allocation, allocation by days of the week or odd-even study numbers) and the outcomes from each group are compared.

**Randomised controlled trial** – the unit of experimentation (eg. people, or a cluster of people[[1]](#footnote-2)) is allocated to either an intervention (the factor under study) group or a control group, using a random mechanism (such as a coin toss, random number table, computer-generated random numbers) and the outcomes from each group are compared. Cross-over randomised controlled trials – where the people in the trial receive one intervention and then cross-over to receive the alternate intervention at a point in time – are considered to be the same level of evidence as a randomised controlled trial, although appraisal of these trials would need to be tailored to address the risk of bias specific to cross-over trials.

**Reference standard** - the reference standard is considered to be the best available method for establishing the presence or absence of the target condition of interest. The reference standard can be a single diagnostic method, or a combination of methods. It can include laboratory tests, imaging tests, and pathology, but also dedicated clinical follow-up of individuals (Bossuyt et al 2003).

**Screening intervention** – a screening intervention is a public health service in which members of a defined population, who do not necessarily perceive that they are at risk of, or are already affected by a disease or its complications (asymptomatic), are asked a question or offered a test, to identify those individuals who are more likely to be helped than harmed by further tests or treatment to reduce the risk of a disease or its complications (UK National Screening Committee, 2007). A screening intervention study compares the implementation of the screening intervention in an asymptomatic population with a control group where the screening intervention is not employed or where a different screening intervention is employed. The aim is to see whether the screening intervention of interest results in improvements in patient-relevant outcomes eg survival.

**Study of diagnostic yield** – these studies provide the yield of diagnosed patients, as determined by the index test, without confirmation of the accuracy of the diagnosis (ie. whether the patient is actually diseased) by a reference standard test.

**Systematic review** – systematic location, appraisal and synthesis of evidence from scientific studies.

**Test** - any method of obtaining additional information on a person’s health status. It includes information from history and physical examination, laboratory tests, imaging tests, function tests, and histopathology (Bossuyt et al 2003).

**Two or more single arm study** – the outcomes of a single series of people receiving an intervention (case series) from two or more studies are compared. *Also see entry on unadjusted indirect comparisons.*

**Unadjusted indirect comparisons** – an unadjusted indirect comparison compares single arms from two or more interventions from two or more separate studies via the use of a common reference ie A versus B and B versus C allows a comparison of A versus C but there is no statistical adjustment for the common reference (B). Such a simple indirect comparison is unlikely to be reliable (see Song et al 2000).

**References relating to explanatory notes and glossary**:

Bandolier editorial. Diagnostic testing emerging from the gloom? *Bandolier*, 1999;70:70-5. Available at: <http://www.jr2.ox.ac.uk/bandolier/band70/b70-5.html>

Bossuyt PM, Reitsma JB, Bruns DE, Gatsonis CA, Glasziou PP, Irwig LM, Lijmer JG, Moher D, Rennie D, de Vet HCW for the STARD Group. Towards complete and accurate reporting of studies of diagnostic accuracy: the STARD initiative. *AJR*, 2003; 181:51-56

Bucher HC, Guyatt GH, Griffith LE, Walter SD. The results of direct and indirect treatment comparisons in meta-analysis of randomized controlled trials. *J Clin Epidemiol*, 1997;50:683-91.

Elwood M. (1998) *Critical appraisal of epidemiological studies and clinical trials*. Second edition. Oxford: Oxford University Press.

Glasziou P, Irwig L, Bain C, Colditz G. (2001) *Systematic reviews in health care. A practical guide.* Cambridge: Cambridge University Press.

Lijmer JG, Mol BW, Heisterkamp S, Bonsel GJ, Prins MH, van der Meulen JHP, Bossuyt PMM. Empirical evidence of design-related bias in studies of diagnostic tests. *JAMA*, 1999; 282(11):1061-6.

Medical Services Advisory Committee (2005). *Guidelines for the assessment of diagnostic technologies*. [Internet] Available at: www.msac.gov.au

Mulherin S, Miller WC. Spectrum bias or spectrum effect? Subgroup variation in diagnostic test evaluation. *Ann Intern Med*, 2002;137:598-602.

NHMRC (1999). *A guide to the development, implementation and evaluation of clinical practice guidelines*. Canberra: National Health and Medical Research Council.

NHMRC (2000a). *How to review the evidence: systematic identification and review of the scientific literature*. Canberra: National Health and Medical Research Council.

NHMRC (2000b). *How to use the evidence: assessment and application of scientific evidence*. Canberra: National Health and Medical Research Council.

Phillips B, Ball C, Sackett D, Badenoch D, Straus S, Haynes B, Dawes M (2001). *Oxford Centre for Evidence-Based Medicine levels of evidence (May 2001)*. Oxford: Centre for Evidence-Based Medicine. Available at: http://www.cebm.net/levels_of_evidence.asp

Sackett DL, Haynes RB. The architecture of diagnostic research. *BMJ*, 2002;324:539-41.

Song F, Glenny A-M, Altman DG. Indirect comparison in evaluating relative efficacy illustrated by antimicrobial prophylaxis in colorectal surgery. *Controlled Clinical Trials*, 2000;21(5):488-497.

UK National Screening Committee (2000). *The UK National Screening Committee’s criteria for appraising the viability, effectiveness and appropriateness of a screening programme*. In: Second Report of the UK National Screening Committee. London: United Kingdom Departments of Health. Pp. 26-27. Available at: <http://www.nsc.nhs.uk/>

Whiting P, Rutjes AW, Reitsma JB, Bossuyt PM, Kleijnen J. The development of QUADAS:a tool for the quality assessment of studies of diagnostic accuracy included in systematic reviews. *BMC Med Res Methodol* 2003; 3(1): 25. Available at: http://www.biomedcentral.com/1471-2288/3/25

1. Known as a cluster randomised controlled trial [↑](#footnote-ref-2)
